# ERRATUM

**DOI:** 10.1590/1678-77572014er001

**Published:** 2016

Due to a publishing error the article “Erosive cola-based drinks affect the bonding to enamel surface: an in vitro study”, published at Journal of Applied Oral Science 22(5):435-442 was printed with the following errors:

Page 435

Material and Methods: “Forty-six bovine enamel specimens…” should be read “Fifty-six bovine enamel specimens…”.

Light Coke “…tritability=12 mL (0. N NaOH).”

*should be read*

Light Coke “…tritability=12 mL (0.1 N NaOH).”

